# Analysis of full-text publication and publishing predictors of abstracts presented at an Italian public health meeting (2005–2007)

**DOI:** 10.1186/s13104-015-1463-7

**Published:** 2015-09-29

**Authors:** S. Castaldi, M. Giacometti, W. Toigo, F. Bert, R. Siliquini

**Affiliations:** Post Graduated School in Public Health, University of Milan, Milan, Italy; Department of Public Health, Post Graduated School in Public Health, University of Turin, Via Santena 5/bis, 10126 Turin, Italy; Department of Public Health, University of Turin, Turin, Italy

**Keywords:** Scientific Societies, Publications, Conferences and congresses

## Abstract

**Background:**

In Public Health, a thorough review of abstract quality evaluations and the publication history of studies presented at scientific meetings has never been conducted. To analyse the long-term outcome of quality abstracts submitted to conferences of Italian Society of Hygiene and Public Health (SItI) from 2005 to 2007, we conducted a second analysis of previously published material aiming to estimate full-text publication rate of high quality abstract presented at Italian public health meetings, and to identify predictors of full-text publication.

**Methods:**

The search was undertaken through scientific databases and search engines and through the web sites of the major Italian journals of Public Health. For each publication confirmed as a full text paper, the journal name, impact factor, year of publication, gender of the first author, type of study design, characteristics of the results and sample size were collected.

**Results:**

The overall publication rate of the abstracts presented is 23.5 %; most of the papers were published in Public Health journals (average impact factor: 3.007). Non universitary affiliation had resulted in a lower probability of publication, while some of the Conference topics had predisposed the studies to an increased likelihood of publication as well as poster form presentation.

**Conclusions:**

The method presented in this study provides a good framework for the evaluation of the scientific evidence. The findings achieved should be taken into consideration by the Scientific Societies during the contributions selection phase, with the aim of achieving a continuous improvement of work quality. In the future, it would be interesting to survey the abstract authors to identify reasons for unpublished data.

## Background

In the international literature, few Medical Scientific Societies and Associations performed quality evaluations of the studies presented at their scientific meetings, but some studies have investigated distinct aspects, such as positive outcome or institutional bias, associated with acceptance at scientific meetings [1–4]; however, none of these associations, neither in Italy nor in other countries, is a Public Health Organization [5–8]. Moreover, most of the available papers, in addition to the abstract quality assessment, are involved in analysing the long term outcome and publication history of the works presented to congress or conferences, with the final aim of identifying factors predicting full publication [9–22]. Even this type of qualitative analysis has never been conducted in the Public Health field.

Moreover, some elements of the scientific data selection process remained unclear. Thus, with the aim of improving the understanding of the pathway of scientific data from congress documents to scientific evidence, some systematic reviews have been conducted [23–25].

Von Elm et al. [25] concluded that approximately one-third of abstracts submitted to biomedical meetings are eventually published as full reports. He identified five factors that possibly play a role in subsequent publication: abstracts that reported on a positive study outcome, abstracts that reported basic research, abstracts presented at meetings with a selected number of participants and abstracts submitted to United States meetings. Using survival-type analysis, he estimated that 27 % were published after 2, 41 % after 4 and 44 % after 6 years.

In Italy, there are few studies of this issue and none in the Public Health field. Vecchi et al. [26] focused on the abstracts’ results and their association with the full publication of contributions presented at the Annual Meeting of College on Problems of Drug Dependence; they concluded that 62 % of the abstracts were subsequently published in peer reviewed journals and that studies with positive findings were more likely to be published.

Considering these data, there is a clear need to provide to public health professional an objective analysis of the potential possibilities and achievements of the evidence discussed during a Public Health Meeting.

The Congress of the Italian Society of Hygiene (SItI) appears to be, in the Italian context, an essential moment at which scientific knowledge is made available to the scientific community, an opportunity for participants to gain experience and an important step in scientific progress. Indeed, these events promote and facilitate collaboration between research groups, and the results obtained from the Congress works are often used in decision making by all Public Health professionals.

Considering the important role played by these conferences in the dissemination of knowledge, in recent years, there has emerged a strong need to submit all the contributions sent as oral communication or posters to an evaluation process, with the aim of analysing the main characteristics and quality of work accepted and then published in the Abstract Books from 2005 to 2010. Castaldi et al. [27] developed an evaluation tool, and the results showed that the average score among all the abstracts reviewed was good. Oral communications showed an average score higher than posters, and according to the affiliation, the highest scores were associated with Universities.

Starting from the results presented by the study mentioned above [27], we have deepened our analysis to analyse more specifically the long-term outcome of good quality abstracts submitted to SItI conferences over a 3-year period (from 2005 to 2007).

Our main objectives are to estimate full-text publication rate of high quality abstract presented at Italian public health meetings, and to identify predictors of full-text publication.

## Methods

During a previous study [27], a total of 4399 abstracts presented to SItI congresses or conferences from 2005 to 2010 were analysed. As reported in this previous article, the reviewers were 11 students from the Postgraduated School in Public Health of the Universities of Turin and Milan, under the supervision of their two School Directors. The amount of agreement within the eight individual criteria of the evaluation checklist was measured by Intraclass Correlation Coefficient (ICC) [27].

The evaluation used eight items related to coherency, structure, originality of the study, definition of study objectives, definition of the type of study, description of data sources, description of results, and conclusions, discussion and practical implications of the study.

For each item, the researcher could assign marks from 0 to 3, so the maximum total marks for each form was 24.

Among all abstracts, only the ones evaluated as “good quality works” were selected for the present study (N = 621). This group includes not only papers with a total score equal to or greater than 19 but also papers with a lower score (between 16 and 18) that scored well in all the items analysed but were not evaluated on one specific item (the “Inherence” item) because it belonged to the miscellaneous topic group. In this regard, the categories of topics were identified according the congress sessions groups, when available. If the themes of the sessions were not available (i.e. for the abstracts accepted as posters in 2005) we classified by a manual revision the abstracts according to the congress sessions of the other years. Following this strategy we identified 11 categories: Food and Nutrition; Health Education; Organization; Vaccines; Epidemiology of Infective Diseases; Epidemiology of Chronic Degenerative Diseases; Environment; Hospital Hygiene; Miscellaneous; Dental Hygiene. Only in the case of Abstracts relating to different subjects but not attributable to previous specific groups we decided to put them in the ‘Miscellaneous’ group.

After a pilot study, the publication history of each abstract presented during 2005, 2006 and 2007 meetings was determined in July 2012, enabling at least a 5-year follow-up. The search was undertaken through PubMed, MEDLINE, the Cochrane library and the web sites of the major Italian journals of Public Health and Hygiene, with no language restrictions. In order to find further papers not published on the previous databases, we decided to include Google Scholar, despite its relative limited scientific value, in our search strategy.

The first search criterion was the combination of the first author’s name and keywords available in the title or abstract. When this search strategy did not identify any publications, to minimise errors in the follow-up, various combinations of words taken in the title and abstract, keywords and author names were tested.

The abstract was considered “published” if at least one author of the abstract was an author of the full publication and the main outcome from the abstract was an outcome in the full manuscript. A change in the sample size, the title or the name or order of some authors or minor changes to the objectives was not considered as a de novo study, whereas manuscripts describing different endpoints were considered as such.

For each publication confirmed as a full-text paper, the journal name, its impact factor and the year of publication were collected.

In the case of abstracts published more than once, we used the earliest publication. Abstracts published in full before the presentation at the Conference were excluded.

In addition, for each abstract of the sample, the following information was collected: gender of the first author (through web search engines), type of study design (experimental, observational descriptive, observational analytical, revision), characteristics of the results (positive or negative) and sample size (n ≤ 100 or n > 100).

All the analysis was performed using STATA-MP 11 software. We performed a descriptive statistical analysis to describe the publication history and the main characteristics of the sample.

All the abstracts characteristics that were available in the conferences databases were included, in particular: affiliation, topics, year, abstract, geographic area, first author gender, study design, results, sample size, total score. It was not performed a preventive selection of the characteristics included.

Then, a univariate logistic regression analysis was performed to test the strength of the associations hypothesised and, finally, the variables associated with a positive outcome of publication (accepted level of statistical significance: p < 0.25, according to the Hosmer–Lemeshow test) were included in a model of multivariate analysis, with the aim of identifying possible factors predicting publication and to remove any confounders [28]. We included the following variables: affiliation, topics, abstract, first author gender, study design, results, total score.

## Results

Among the 4399 abstracts accepted from 2005 to 2007 by the SItI for its annual conference, only 621 abstracts were included in the analysis (31.6 %), meeting the main inclusion criteria of the study.

The main descriptive results are shown in Table 1. Most of the works were presented in 2007 (41.9 %), 30.1 % in 2005 and 28 % in 2006. Considering all the 3 years in study, the most represented categories of topics are Organization (19.3 %), Health education (15.6 %), Epidemiology of infectious disease (14 %) and Food and Nutrition (10.8 %). The more frequent affiliation was University (68 %), followed by Non Universitary Hospitals (15 %). Although the works were all selected for their good quality, it was decided to split them into three groups according to the total score previously achieved.

**Table 1:** Baseline characteristics of abstract evaluated (N = 621)

	%
Abstract	
Poster	76.0
Oral	24.0
First author gender	
Female	51.5
Male	48.5
Affiliation	
University	68.0
Non Universitary hospitals	15.0
Other	11.7
Study design	
Local Public Health Institution	5.3
Observational analytical	41.8
Observational descriptive	41.0
Sperimental	10.2
Revision	7.0
Results	
Positive	72.6
Not evaluable	20.8
Negative	6.6

Thirty per cent of the papers reached a score between 16 and 18 (medium quality); 52.5 % were high quality works that had a score between 19 and 21, while only 17.5 % could be defined as very high quality works with a score between 22 and 24.

By considering the main outcome of the study (Table 2), it can be noted that the overall publication rate of the abstracts presented is 23.5 % and that most of the papers were published in Public Health journals (53.4 %). Among all the journals, 63 % were peer reviewed, and the impact factor goes from 0.441 to 6.600 with an average value of 3.007.

**Table 2:** Publication rates and publication history of the evaluated abstract (N = 621)

	2005	2006	2007	Total sample
Publication rate	22.5 %	23.6 %	24.2 %	23.5 %
Journal type				
Public health	38.1 %	58.5 %	60.3 %	53.4 %
Other	61.9 %	41.5 %	39.7 %	46.6 %
Peer reviewed journal	66.7 %	58.5 %	63.5 %	63.0 %
Average impact factor	2.75 CI (2.20–3.29)	3.27 CI (2.74–3.79)	3.03 CI (1.48–2.36)	3.00 CI (2.63–3.43)
Average time of publication gap (years)	2.28 CI (1.73–2.83)	2.54 CI (2.05–3.01)	1.68 CI (1.38–1.98)	2.09 CI (1.84–2.34)

The average time gap between the presentation at the SItI Conferences and the publication in full text was 2.1 years.

Table 3 shows the characteristics of the papers published in full according to the most cited variables predictive of publication [16, 17, 19, 28, 29].

**Table 3:** Full text publication vs unpublished papers (N = 621)

	Full text publication		Unpublished		p value
	N	%	N	%	
Affiliation					
University	120	28.4	302	71.6	<0.005
Non Universitary hospitals	3	3.2	90	96.8	
Local Public Health Institution	5	15.2	28	84.8	
Other	18	24.7	55	75.3	
Topics					
Food and nutrition	15	22.4	52	77.6	<0.005
Environment	13	27.7	34	72.3	
Health education	21	21.6	76	78.4	
Chronic disease	20	37.0	34	63.0	
Infectious disease	16	18.4	71	81.6	
Dental hygiene	5	71.4	2	28.6	
Hospital hygiene	9	15.8	48	84.2	
Miscellaneous	13	26.0	37	74.0	
Organization	17	14.2	103	85.8	
Vaccine	17	48.6	18	51.4	
Year					
2005	42	22.5	145	77.5	0.909
2006	41	23.6	133	76.4	
2007	63	24.2	197	75.8	
Abstract					
Oral	54	36.2	95	63.8	<0.005
Poster	92	19.5	380	80.5	
Geographic area					
Northern Italy	53	25.1	158	74.9	0.568
Central Italy	60	21.5	219	78.5	
Southern Italy	33	25.2	98	74.8	
First author gender					
Male	62	20.7	237	79.3	0.216
Female	84	26.4	234	73.6	
Study design					
Sperimental	21	33.3	42	66.7	0.051
Observational descriptive	47	18.5	207	81.5	
Observational analytical	67	25.9	192	74.1	
Revision	11	25	33	75	
Results					
Negative	4	9.8	37	90.2	<0.005
Positive	121	26.9	329	73.1	
Not evaluable	21	16.3	108	83.7	
Sample size					
≤100	47	21.4	173	78.6	0.342
>100	99	24.7	301	75.3	
Total score					
Medium	30	16.1	156	83.9	<0.005
High	78	24.0	248	76.0	
Very high	38	35.0	71	65.0	

The University affiliation was more associated with the publication in full text, as were some Conference topics (48.6 % Vaccine, 37 % Chronic disease).

The study design (33.3 % of the experimental studies and 25.8 % of the observational analytical ones) and the characteristics of the results (9.8 % of the studies with negative results and 26.9 % of those with positive results) seem to be associated with the likelihood of being published.

Furthermore, the increase of the quality score assigned to the works during the evaluation phase seems to be a characteristic more associated with the subsequent publication. All these associations were supported by statistical significance (p < 0.005). The results related to the potential highest rate of publication by females (26.4 vs 20.7 %; p = 0.216) and by sample size (24.7 % of the studies with n > 100 were published in extenso compared to 21.4 % of the studies with n ≤ 100; p = 0.342) were, instead, no statistically significant.

With the aim of testing the strength of all these associations, we carried out a univariate linear regression. Through this type of analysis, we investigated the single association between each variable and the main outcome of the study: the publication of the works in extenso.

The variables associate, with an accepted level of statistical significance (p < 0.25, according to the Hosmer–Lemeshow test), with a positive outcome of publication in a model of multivariate analysis. The results are shown in Table 4.

**Table 4:** Multivariate analysis. Factors predicting publication (N = 621)

	Multivariate regression			
	N	Odds ratio	95 % CI	P value
Affiliation				
University	120	1	–	–
Local Public Health institution	5	0.64	(0.22–1.85)	0.418
Non universitari Hospitals	3	0.09	(0.03–0.33)	<0.001
Other	18	0.91	(0.49–1.70)	0.773
Topics				
Food and nutrition	15	1	–	–
Environment	13	1.24	(0.50–3.09)	0.638
Health education	21	0.94	(0.42–2.13)	0.897
Chronic disease	20	1.91	(0.80–4.60)	0.145
Infectious disease	16	0.78	(0.34–1.80)	0.564
Dental hygiene	5	10.52	(1.47–75.18)	0.019
Hospital hygiene	9	0.77	(0.29–2.04)	0.600
Miscellaneous	13	1.73	(0.67–4.42)	0.254
Organization	17	0.71	(0.31–1.63)	0.419
Vaccine	17	3.45	(1.32–9.00)	0.011
Abstract				
Oral	54	1	–	–
Poster	92	0.60	(0.37–0.99)	0.044
First author gender				
Male	62	1	–	–
Female	84	1.31	(0.86–1.99)	0.212
Study design				
Sperimental	21	1	–	–
Observational descriptive	47	0.74	(0.36–1.52)	0.415
Observational analytical	67	1.08	(0.56–2.08)	0.820
Revision	11	1.12	(0.43–2.93)	0.820
Results				
Negative	4	1	–	–
Positive	121	3.43	(1.03–11.4)	0.044
Not evaluable	21	2.23	(0.61–8.17)	0.228
Total score				
Medium	30	1	–	–
High	78	1.60	(0.94–2.72)	0.086
Very high	38	2.72	(1.39–5.31)	0.003

The Non Universitary Hospitals affiliation, in comparison with the University one, results in a lower probability of publication. This finding does not change in the multivariate analysis, with a corrected odds ratio of 0.09 (p < 0.001).

Moreover, the analysis revealed some topics that predispose the studies to a statistically significant increased likelihood of publication, such as Dental hygiene (OR 10.52, but the abstracts related to this topic were only 7) and Vaccine (OR 3.45).

The first author female gender is confirmed to be associated with an increased likelihood of publication (adjusted OR 1.31), but this association is not statistically significant (p = 0.212).

Similarly, the association between a higher probability of publication and the typology of the study shows an advantage of the experimental designs over the descriptive observational studies (adjusted OR 0.74), but the statistical significance (p = 0.011) revealed in the univariate analysis is not confirmed in the multivariate one.

Regarding the abstract quality score, a positive trend emerges: a high evaluation score means there is a higher probability the work will be published in extenso (p = 0.003).

## Discussion

As the SItI conferences represent a fundamental moment in the Italian Public Health field, we think an evaluation of potential predictors of publication on the international literature of studies presented in these meetings can represent a due starting point for suggesting improvements.

In regard to the publication rate, from the analysis, it emerged that 23.5 % of the high quality abstracts presented at SItI conferences were subsequently published in the literature. This value is lower compared to other studies: for example, Winnik et al. [19] indicated a publication rate of 38 %, and Raptis et al. [20] indicated a rate of 40 %. However, our value is similar to the Yoon et al. [16] rate (30 %) and to the Chand et al. [18] rate (30 %). As example, the study of Chand et al. [18] retrieved all abstracts from the Scientific Meetings of the Cardiac Society of Australia and New Zealand from 1999 to 2005. Only 30 % of the 2172 abstracts were followed by publication of a full-text article, and most publications were published within 1 (61 %) or 2 years (84 %).

Such diversity could be related to the differences in the study designs. In the clinical field, there are more frequent randomised clinical trials, which are subsequently published more easily than observational studies. As example, in regard to the surgical field, Raptis et al. [20] conducted an evaluation to assess the peer review process of the European Surgical Association from 2002 to 2007. Approximately one-third of the contributions were accepted for presentation at the annual meetings and, of those, 40 % were published in Annals of Surgery. The authors found, accordingly with the previous hypothesis, only two independent factors able to promote subsequent publication: randomised controlled trials as the study design and a sample size with more than 100 patients.

Other good quality abstracts do not reach publication, in our opinion, for logistical or qualitative reasons. Logistical reasons could be due to various possibilities: (1) “Lack of time” for the preparation of a full manuscript text (e.g., for professionals employed in non universitary hospitals). (2) Losing confidence when results are not clinically or statistically significant [22, 29]. (3) Other papers have similar findings. Qualitative reasons include inadequate study design, methodology or grammatical style, including language barriers, which may prevent the work from surviving the peer review process.

Our results are different than those of Gorman et al. [11], who concluded that only 36 % of abstracts presented in Toxicology Meetings were published in peer review journals.

Regarding the overall mean impact factor, the Yoon et al. study [16] reported a value for published research of 2.90. Thus, the overall publication rate was relatively low compared not only with other urological meetings held in America and Europe but also with the SItI Conferences.

Conversely, Winnik et al. [19] indicated that the works presented to the European Society of Cardiology Congress reached very high impact factor values: approximately 40 % of the abstracts were placed over 5. In this case, however, the types of works presented include Randomized Clinical Trials, meta-analyses and systematic reviews that are almost absent in our sample and the fact that Public Health Journals have, usually, a lower IF than clinical ones.

The distribution of time to publication for abstracts was consistent with previous studies of publication, occurring within 2–3 years [16, 17, 30, 31].

The analyses revealed a significant disadvantage for non-university-affiliated institutions. The reasons behind this difference may result from a greater willingness and ability of academic professionals compared to hospital ones in conducting and directing the different steps that range from abstract to publication. It must be noted that this result is in agreement with the conclusions reached by other authors [19, 32]. Winnik et al. [19], as example, performed a 4-year follow-up of the abstracts submitted to the European Society of Cardiology Congress in 2006 in order to identify factors predicting high-quality research. In their study they found that 38 % of all accepted studies were subsequently published and that the presence of an academic affiliation and a prospective study design were associated with full-text publication.

Moreover, from the analysis, it emerges that certain Conference topics predispose the studies to an increased likelihood of publication. This result can be partially explained by the fact that both the topics (Dental hygiene and Vaccine) are, on one hand, more subjected to clinical trial and, on the other, not strictly related to national settings.

Regarding oral presentation, most authors did not analyze this item [5, 18] or because the study design [10, 11, 13] or because they were not able to distinguish whether the study was presented as a poster or podium presentation [16]. Winnik [19] analyzed the abstracts presentation type but did not find any statistical correlation. Otherwise, according to our findings, Krzyzanowska [9] found that studies with oral or plenary presentation were published sooner than those not orally presented (p = 0.002) and also Schnatz [17] wrote that the average time to publication for oral presentations was 1.7 ± 1.3 years, while for poster presentations was 2.0 ± 1.5 years (P = 0.241). The publication rate of oral presentations was significantly higher than the poster presentations rate (57.7 vs 36.5 %; P < 0.003).

We may assume that the research that is presented orally may be judged by the reviewers as having greater interest and clinical relevance along with more sound methodology and better results.

In the literature, few authors have analysed how gender could affect the success of authors submitting posters or abstracts [19].

Interestingly, the rate of full-text publication of male authors seemed lower compared with their female colleagues (20.7 vs. 26.4 %), but in the multivariate analysis no statistical significance was found for the gender in predicting full-text publication.

Our results differ from those of Winnik et al. [19] in that, in the cardiology field, the female gender was identified as a factor that negatively affects scientific success.

Of course, all of the above findings should be interpreted cautiously and considered exploratory. The importance of understanding the role of gender in research is critical and certainly requires further consideration.

No statistically significant differences were identified regarding the study designs of abstracts included in our analysis. This result is quite interesting, considering the peculiarities of public health field, where very often the papers published are not comprehensive of numeric data but instead related to organizational perspectives or policies discussion.

Abstracts that claim to have achieved results positive and consistent with objectives are more likely to be published (adjusted OR 3.43). This result might suggest that scientific journals tend to prefer works with positive results or that authors themselves are inclined to send such works to editors, making a selection a priori and focusing on more appealing studies. These types of behaviours certainly promote the publication bias.

Regarding the abstract quality score, a positive trend emerges: with the score increasing, there is a higher probability that the work is published in extenso. This result shows that the evaluation method applied has a high degree of agreement with the scientific journal editors’ opinions and judgments.

### Limitations and further studies

This study has some limitations that deserve discussion. First of all, our search algorithm could potentially miss some papers that may have been published in journals not listed on Medline. It is known that Medline lists up to 80 % of the total journal articles published worldwide [33]. Moreover, if the authors, the title or the hypothesis of the study were substantially modified during the process of editing and supplementing the data, our algorithm may have not detected the article in our search. However, we tried to limit this phenomenon by performing very thorough research.

A potential limitation is represented by the choice to include only high quality abstracts in our analysis. However, we declared this selection strategy as main inclusion criteria in the aim and in the methods section of the study.

## Conclusions

Authors have an ethical obligation to endeavour in the distribution of their original findings through scientific publication, consequently improving the quality of scientific research. Once available to the public and to other health professionals, this research can be followed up and implemented in the best interest of the patient [34].

To make a useful and precise selection, it is necessary to know the main features related to the publication, and the data presented in this study provide a good framework.

It would be interesting, through further research, to survey the abstract authors to identify reasons for unpublished data and to learn what percentage is due to logistical versus qualitative reasons. As part of that follow-up, analysis of funding type, the country from which the research originated, pharmaceutical company involvement or support, clinical versus laboratory studies or other potential biases for publication could be assessed to evaluate whether they affect either the likelihood of or time to publication. Insight into reasons for delays and the number of submissions until publication would also be informative.
